# Effects of Saline-Alkaline Stress on Metabolome, Biochemical Parameters, and Histopathology in the Kidney of Crucian Carp (*Carassius auratus*)

**DOI:** 10.3390/metabo13020159

**Published:** 2023-01-20

**Authors:** Lu Ding, Yingjie Liu, Xiaofeng Wei, Chuanye Geng, Wenzhi Liu, Lin Han, Fangying Yuan, Peng Wang, Yanchun Sun

**Affiliations:** 1 Laboratory of Quality & Safety Risk Assessment for Aquatic Products, Heilongjiang River Fisheries Research Institute, Chinese Academy of Fishery Sciences, Ministry of Agriculture and Rural Areas, Harbin 150070, China; 2 Department of Food Science and Engineering, College of Food Science and Technology, Shanghai Ocean University, Shanghai 201306, China; 3 Department of Food Science and Engineering, College of Food Science and Engineering, Dalian Ocean University, Dalian 116023, China; 4 Department of Chemical Engineering and Technology, College of Materials and Chemical Engineering, Harbin University of Science and Technology, Harbin 150080, China

**Keywords:** saline-alkaline stress, crucian carp, kidney, metabolomics, histopathology

## Abstract

The salinization of the water environment caused by human activities and global warming has increased which has brought great survival challenges to aquatic animals. Crucian carp (*Carassius auratus*) is an essential freshwater economic fish with superior adaptability to saline-alkali water. However, the physiological regulation mechanism of crucian carp adapting to saline-alkali stress remains still unclear. In this study, crucian carp were exposed to freshwater or 20, 40, and 60 mmol/L NaHCO_3_ water environments for 30 days, the effects of saline-alkali stress on the kidney were evaluated by histopathology, biochemical assays and metabolomics analysis from renal function, antioxidant capacity and metabolites level. Our results showed different degrees of kidney damage at different exposure concentrations, which were characterized by glomerular atrophy and swelling, renal tubular degranulation, obstruction and degeneration, renal interstitial edema, renal cell proliferation and necrosis. Saline-alkali stress could change the levels of several physiological parameters with renal function and antioxidant capacity, including creatinine (CREA), urea nitrogen (BUN), superoxide dismutase (SOD), catalase (CAT), glutathione peroxidase (GSH-Px) and malondialdehyde (MDA). In addition, metabolomics analysis showed that differential metabolites (DMs) were involved in various metabolic pathways, including phenylalanine, tyrosine, and tryptophan biosynthesis, aminoacyl-tRNA biosynthesis, purine metabolism, glycerophospholipid metabolism, sphingolipid metabolism, glycolysis/gluconeogenesis and the TCA cycle. In general, our study revealed that saline-alkaline stress could cause significant changes in renal function and metabolic profiles, and induce severe damage in the crucian carp kidney through destroying the anti-oxidant system and energy homeostasis, inhibiting protein and amino acid catabolism, as well as disordering purine metabolism and lipid metabolism. This study could contribute to a deeper understanding the adverse effects of saline-alkali stress on crucian carp kidney and the regulatory mechanism in the crucian carp of saline-alkali adaptation at the metabolic level.

## 1. Introduction

Globally, aquatic products provide nearly 20% of the daily average protein intake for people [1] and make essential contributions to improving the dietary structure of residents and ensuring world food security [2]. China is a major country in aquaculture in the world, of which aquaculture output is more than 60 % of the total output of aquaculture in the world [3]. There are about 4.6 × 10^11^ m^2^ of low-lying saline-alkali waters in China [4], most of which remain unexploited due to the special water quality conditions of significant carbonate alkalinity (CA) and ion imbalances [5]. CA is considered a crucial stress factor as it affects the survival of aquatic animals in saline-alkali water [6]. Previous studies have shown that carbonate alkalinity could cause a series of problems for aquatic animals, such as respiratory and metabolic alkalosis [7], tissue oxidative damage [8], intestinal flora imbalance [9], as well as disorders of the antioxidant and immune system [10], which seriously affect the normal growth, development and reproduction of fish. At the same time, recent studies have shown that the extent of freshwater salinization continues to deepen as a result of human activities and global warming, which might steadily shrink the available space for freshwater aquaculture [11,12]. Despite all this, there are still some salt-tolerant dominant fish species such as crucian crap (*Carassius auratus*) [13], amur ide (*Leuciscus waleckii*) [14], and scale-less carp (*Gymnocypris przewalskii*) [15], which have developed behavioral and physiological adaptations to survive in saline-alkaline water. Therefore, it is very important to master the response regulation mechanism of freshwater fish saline-alkaline adaptation for cultivating excellent varieties of salt-tolerant fish and promoting the efficient utilization of saline-alkali water.

The teleostean kidney consists of two parts head and body [16]. The body kidney is composed of a large number of glomeruli, renal tubules, collecting tubes and lymph, which not only has hematopoietic and immune functions, but also urinary functions, and play an extremely important role in detoxification, osmotic pressure regulation, acid-base balance and waste excretion [17]. Fish can modulate adaptive plasticity and evolutionary change in kidney function to adapt to saline-alkali stress [18]. Oğuz et al. [19] found that the activity of Na^+^-K^+^-ATPase in the kidney of Van fish (*Alburnus tarichi*) in a saline-alkali lake was significantly lower than that in a freshwater lake, and the glomerular structure adapted to the saline-alkali lake collapsed, and the lumen of the collecting tube shrank. Mi [15] used cloned gene sequences to find that freshwater *Leuciscus waleckii* responded to the high carbonate alkalinity environment by increasing the gene expression of kidney phosphatase. Chang et al. [20] found that there are a large number of candidate genes for the alkali stress response in the kidney of *Leuciscus waleckii* in a saline-alkali lake by transcriptomics technology. These studies can determine the effects of carbonate alkalinity on the structure, antioxidant system and transcription level in fish kidneys, while failing to provide important information on metabolite levels, making the accurate identification of critical pathways affected by carbonate alkalinity stress challenging.

Crucian carp (*Carassius auratus*) is an important fish species of freshwater aquaculture in China. It has the characteristics of fast growth, and high-stress resistance, which is a reliable model for studying the physiological mechanism of saline-alkali tolerance of freshwater fish [21]. Recently, metabolomics reveal the metabolic essence and microenvironment state of organisms by capturing the changes in the metabolic characteristics of endogenous and exogenous small molecules with systematic biological methods [22]. It has been widely applied in unraveling underlying mechanisms of physiological processes and abiotic stress responses in aquatic organisms [23]. Previous studies have reported the metabolic variation characteristics of the crucian carp gill and liver in response to saline-alkali stress by using metabolomics technology [21,24,25]. However, there is little information concerning the mechanism underlying the carbonate alkaline stress-induced toxic effects and changes in metabolic profile.

In the present study, we first studied the effects of different concentrations of NaHCO_3_ exposure on crucian carp kidney morphology. Meanwhile, classical biochemical indices were measured to evaluate the antioxidant capacity and renal function of crucian carp kidneys after carbonate alkalinity exposure. A UPLC-QTOF/MS metabolomics strategy was then systematically applied to identify different concentrations of NaHCO_3_ exposure-induced changes in metabolites. Our study would provide new insights into the molecular mechanisms behind the adverse effects of saline-alkaline water on freshwater fish kidneys and the coping strategies of freshwater fish to carbonate alkalinity stress.

## 2. Materials and Methods

### 2.1. Chemicals and Reagents

Sodium bicarbonate (NaHCO_3_) and xylene were purchased from Aladdin (Shanghai, China). Hematine was purchased from Solarbio (Beijing, China). Eosin Y was purchased from Sangon (Shanghai, China). HPLC grade methanol and acetonitrile were obtained from Merck (Darmstadt, Germany). Formic acid was sought from Anpel (Shanghai, China). Tricaine (MS-222) was obtained from Sigma Aldrich (St. Louis, MI, USA). The detection kits of creatinine (CREA), urea nitrogen (BUN), malondialdehyde (MDA), superoxide dismutase (SOD), catalase (CAT) and glutathione peroxidase (GSH-Px) were acquired from Nanjing Jiancheng Bioengineering Institute (Nanjing, China).

### 2.2. Animal Experimental and Design

Crucian carp were taken from the Hulan Experimental Station of Heilongjiang River Fisheries Research Institute, Chinese Academy of Fisheries Sciences. All the fish were domesticated in fiberglass tanks (200 L, 100 × 50 × 40 cm) containing dechlorinated tap water for two weeks. After acclimatization, a total of 180 healthy and active fish (130.15 ± 3.56 g,) were randomly haphazardly separated into four groups, a control group with dechlorinated water (Con) and three carbonate alkalinity (CA) exposure groups with 20 mmol/L NaHCO_3_ (CA20), 40 mmol/L NaHCO_3_ (CA40), 60 mmol/L NaHCO_3_ (CA60). Each group of 45 individuals included three replicate fiberglass tanks, with 15 fish in each tank. The concentration of CA was determined based on our previous experiment with the data of the high saline-alkaline water areas in aquatic ecosystems in China [22,26]. During the acclimation and experimental periods, the water temperature was 23.0 ± 1.0 °C, dissolved oxygen was greater than 7.5 mg/L, the photoperiod was a natural photoperiod, the experimental fish were fed with commercial food pellets (Tongwei Feed Company, Tianjin, China) twice daily (at 8:00 and 18:00) at 3~5% of their body weight until one day before sampling. One-third of the water in the tanks was exchanged with water of the corresponding CA concentration every day to maintain good water quality.

After 30 days of CA exposure, six fish from each replicate tank were anesthetized with MS-222 (100 mg/L) and dissected in sterile condition to collect kidney tissues and blood samples (18 fish from each group, n = 18). The blood samples were collected from the caudal vein using sterile syringes and sterile centrifuge tubes. A total of 18 kidney samples were collected from each group, of which three were fixed in 4% paraformaldehyde for paraffin sections to observe histological changes. The remaining 15 were stored at −80 °C, 8 for metabolomics analysis and seven for biochemical analysis.

### 2.3. Biochemical Parameters Determination

The blood samples were clotted at 4 °C for 3 h, centrifuged at 4 °C, and 3000 g for 10 min to obtain serum. The levels of serum blood ammonia, CREA and BUN were measured to assess renal function sensitively. The kidney samples (100 mg) were homogenized with 900 μL chilling normal saline for 2 min (60 Hz, −20 °C), and centrifuged for 10 min (2500 g, 4 °C) to collect the supernatant for determining indicators of oxidative injury, including MDA, SOD, CAT and GSH-Px. All biochemical assays were strictly performed according to the manufacturer’s instructions. In this study, seven independent samples from each group were set as biological replicates for the biochemical analyses.

### 2.4. Histopathological Observation

Three fishes from each group were observed histological changes. The fixed kidney samples were dehydrated in ascending grades of ethanol, transparent with xylene, embedded in paraffin, sectioned at 5 μm thickness with a microtome (RM2235, LEICA, Wetzlar, Germany), and stained with hematoxylin and eosin (H&E). Finally, the stained sections of kidney tissue were photographed using an optical microscope (BX53, OLYMPUS, Tokyo, Japan) and photographed under 400× magnification (DP73, OLYMPUS, Tokyo, Japan). The kidney histological changes of the CA stress group were compared with those in the control group.

### 2.5. UPLC-QTOF/MS Analysis

Eight biological replicates for each group were assayed for sample extraction and metabolomics analysis. The metabolite extraction process of the crucian carp kidney was performed according to our previous study [21]. The details regarding metabolomics analysis were described in the Supplementary Materials. Preprocessing of UPLC-QTOF/MS raw data (.wiff) was conducted by Progenesis QI 2.1 software (Waters Corporation, Milford, MA, USA) including peak picking, alignment and normalization, in which logarithmic transformation and the unit variance (UV) scaling algorithm were performed. The parameters were as follows: the minimum intensity was set to 15% of base peak intensity, the noise elimination level was set at 10.00 precursor tolerance was 5 ppm, fragment tolerance was 10 ppm, and retention time (RT) tolerance was 0.02 min. Metabolites identification and annotation were based on MS/MS fragmentation and public databases Human Metabolome Database (HMDB) and Lipidmaps (http://www.hmdb.ca/ (accessed on 15 September 2022), http://www.lipidmaps.org/ (accessed on 15 September 2022)). Then, a processed data matrix including metabolite name, M/Z, retention time, and peak intensities was used as a targeted dataset. The data matrix consisted of 4154 and 3132 metabolites in positive and negative ion modes, respectively (n = 8 replicates per group), which was imported into SIMCA 14.1 software (Umetrics, Umea, Sweden) for principal component analysis (PCA) and orthogonal partial least squares discriminant analysis (OPLS-DA) to determine the metabolic alterations between CA stress and control groups. The differential metabolites (DMs) were selected based on variable importance in the projection (VIP) > 1 of the OPLS-DA model and *p* < 0.05 of the Student’s *t*-test. Metabolic pathways were assessed using MetaboAnalyst 5.0 (https://www.metaboanalyst.ca/ (accessed on 15 September 2022), Xia Lab, Ste. Anne de Bellevue, QC, Canada) and the Kyoto Encyclopedia of Genes and Genomes (KEGG) (https://www.genome.jp/kegg/ (accessed on 15 September 2022)) database.

### 2.6. Statistical Analysis

The difference in biochemical assays between CA stress and control groups was carried out using one-way analysis of variance (ANOVA) through GraphPad Prism 9.0 (GraphPad Software Inc., San Diego, CA, USA). Results were expressed as mean ± SD (n = 7), asterisk (*) indicates that there is a significant difference between CA stress and control groups (* *p* < 0.05, ** *p* < 0.01 and *** *p* < 0.001).

## 3. Results

### 3.1. Biochemical Index Changes after Carbonate Alkalinity Exposure

**Figure 1:**
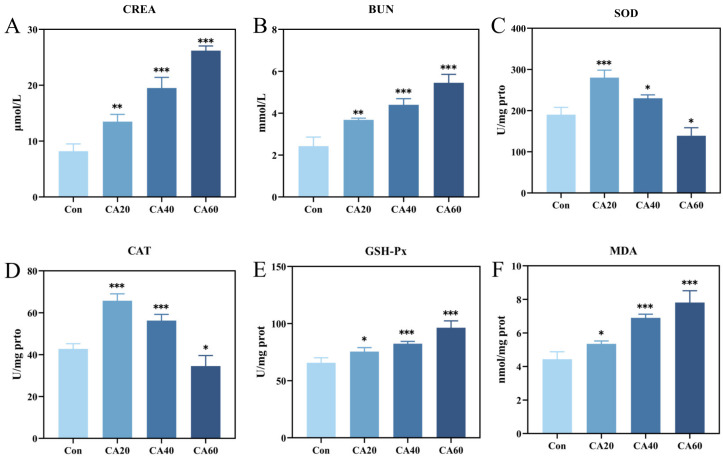
The renal biochemical indexes change of crucian carp under different concentrations of carbonate alkali exposure. (**A**) creatinine (CREA). (**B**) urea nitrogen (BUN). (**C**) superoxide dismutase (SOD). (**D**) catalase (CAT). (**E**) glutathione peroxidase (GSH-Px). (**F**) malondialdehyde (MDA). Con represents the freshwater control group, CA20 represents 20 mmol/L NaHCO_3_ exposure group, CA40 represents 40 mmol/L NaHCO_3_ exposure group, CA60 represents 60 mmol/L NaHCO_3_ exposure group. * *p* < 0.05; ** *p* < 0.01; *** *p* < 0.001.

The changes in renal biochemical indices of crucian carp in the present study after 30-day exposure to CA at different concentrations were displayed in Figure 1. Compared to the freshwater control group, the GSH-Px activity (Figure 1E) and the levels of CREA (Figure 1A), BUN (Figure 1B), and MDA were markedly increased in the CA exposure groups in a concentration-dependent manner. The activities of SOD (Figure 1C) and CAT (Figure 1D) in the CA20 and CA 40 groups increased significantly, while those in the CA60 group decreased significantly.

### 3.2. Histological Changes of the Kidney after Carbonate Alkalinity Exposure

**Figure 2:**
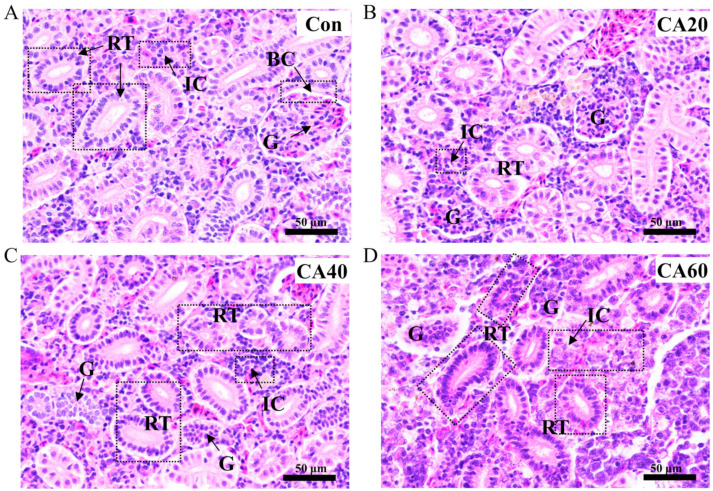
The renal histological alterations of crucian carp under different concentrations of carbonate alkali exposure. (**A**) the freshwater control group: normal histological structure, glomerulus (G), renal tubules (RT), Bowman’s capsules (BC), interstitial cells (IC). (**B**) 20 mmol/L NaHCO_3_ exposure group: glomerular atrophy, renal tubular degranulation and interstitial cell proliferation. (**C**) 40 mmol/L NaHCO_3_ exposure group: glomerular atrophy, renal tubular obstruction and interstitial cell proliferation. (**D**) 60 mmol/L NaHCO_3_ exposure group: glomerular atrophy and swelling with widened Bowman’s capsules, renal tubular degeneration and swelling, renal interstitial edema and interstitial cell necrosis. (H&E, ×400, scale bar: 50 μm).

A series of histopathological alterations were observed in the renal tissues of fish exposed to different concentrations of carbonate alkalinity in Figure 2. In the control group, the renal tubular epithelial cells were arranged neatly, and the glomerulus had a regular shape with intact Bowman’s capsules. In the CA20 group, vacuolation of the renal tubular epithelial cells with tubular constriction, glomerular atrophy and interstitial cell proliferation could be clearly observed. In the CA40 group, there was significant clogging of the renal tubular, interstitial cell proliferation, and glomerular atrophy with thinning Bowman’s capsules. In the CA60 group, we found the following histological changes: interstitial cell necrosis and swelling, glomerular atrophy and swelling with widened Bowman’s capsules, and diffuse degeneration of renal tubular epithelial cells.

### 3.3. Metabolome Alterations of the Kidney after Carbonate Alkalinity Exposure

**Figure 3:**
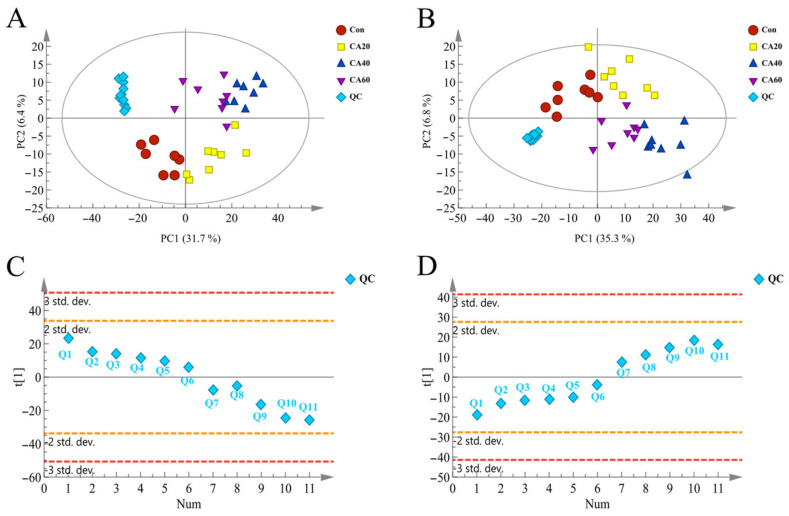
The PCA analysis of crucian carp kidney samples. (**A**) The PCA plot of positive ion mode. (**B**) The PCA plot of negative ion mode. (**C**) The standard deviation of QC samples in positive ion mode. (**D**) The standard deviation of QC samples in negative ion mode. Con represents the freshwater control group, CA20 represents the 20 mmol/L NaHCO_3_ exposure group, CA40 represents the 40 mmol/L NaHCO_3_ exposure group, and CA60 represents the 60 mmol/L NaHCO_3_ exposure group. Orange and red lines indicate the 2 SD and 3 SD limits of peak height intensities, respectively.

**Figure 4:**
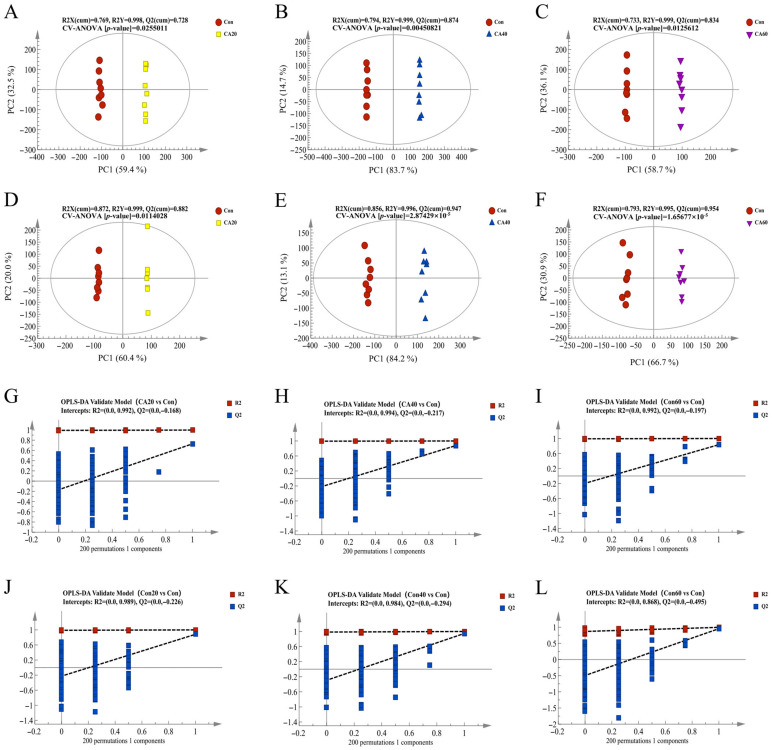
The OPLS-DA analysis of crucian carp kidney under different concentrations of carbonate alkali exposure. (**A**–**C**) The OPLS-DA plot of positive ion mode. (**D**–**F**) The OPLS-DA plot of negative ion mode. (**G**–**I**) The OPLS-DA permutation test of positive ion mode. (**J**–**L**) The OPLS-DA permutation test of negative ion mode. Con represents the freshwater control group, CA20 represents the 20 mmol/L NaHCO_3_ exposure group, CA40 represents the 40 mmol/L NaHCO_3_ exposure group, and CA60 represents the 60 mmol/L NaHCO_3_ exposure group.

The changes in renal metabolites of crucian carp induced by various exposure concentrations of carbonate alkalinity were detected by multivariate statistical pattern recognitions, including PCA and OPLS-DA analysis. The scatter plot of PCA scores for untargeted metabolomics data showed significant separation between the experimental groups, indicating that there were considerable differences in kidney metabolism of crucian carp under different carbonate alkali exposure concentrations (Figure 3). All QC samples were clustered tightly within the ±2 STD range in the PCA plots, and 80% of the metabolites in the QC samples had RSD < 15 %, indicating that the metabolomics platform had high accuracy and reproducibility, and the data were suitable for further analysis. The OPLS-DA model was employed to determine the metabolic difference between CA stress and control groups. As shown in Figure 4A–F, the values of R^2^X, R^2^Y and Q^2^ of the OPLS-DA model quality parameters were higher than 0.8, suggesting that the established OPLS-DA model had a high explained variation and predictive ability. Moreover, the permutation test (n = 200) and CV ANOVA analysis were employed to assess whether the OPLS-DA models were reliable and overfitting; 200 permutation tests of OPLS-DA showed the permuted R^2^ and Q^2^ on the left was lower than the original R^2^ and Q^2^ on the right and Q^2^ was <0, the CV-ANOVA *p*-value for OPLS-DA were all below 0.05. These results demonstrated that OPLS-DA models in both negative and positive ion modes were reliable without overfitting (Figure 4E–L).

**Figure 5:**
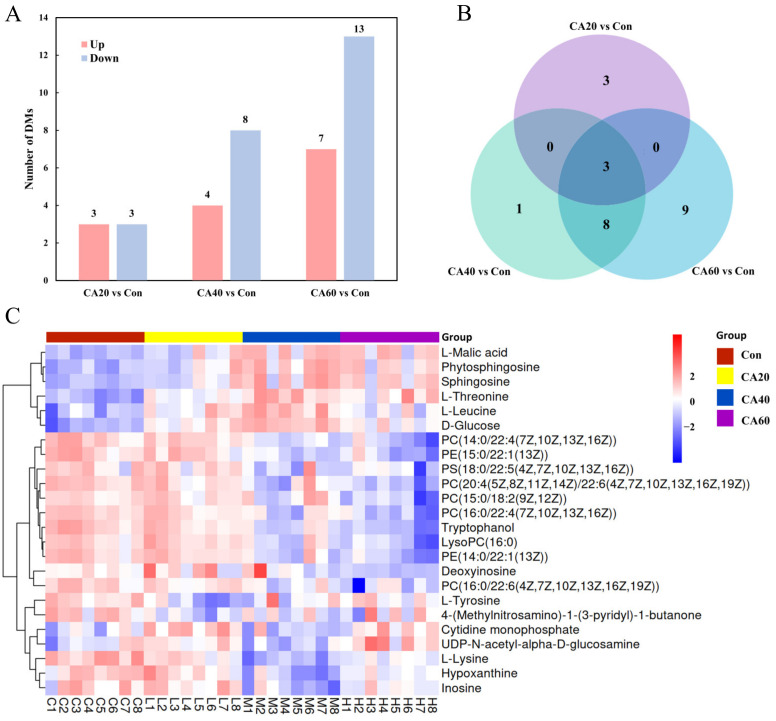
Differential metabolites of crucian carp kidney under different concentrations of carbonate alkali exposure. (**A**) Number of DMs. (**B**) Venn diagram of DMs. (**C**) Cluster heatmap of DMs. Con represents the freshwater control group, CA20 represents the 20 mmol/L NaHCO_3_ exposure group, CA40 represents the 40 mmol/L NaHCO_3_ exposure group, and CA60 represents the 60 mmol/L NaHCO_3_ exposure group.

To better understand the changes in the level of metabolites among different CA stress groups, the metabolites with VIP > 1.0 and *p*-value < 0.05 were identified as differential metabolites (DMs). Compared with the control group, 6, 12, and 20 metabolites were identified as DMs in the kidney exposed to 20, 40, and 60 mmol/L NaHCO_3_, respectively (Figure 5A). The visualized Venn diagram showed the overlay and specificity of the DMs in three CA exposure groups (Figure 5B). To intuitively compare alterations in the levels of metabolites among groups, hierarchical clustering analysis was performed using the Ward.D2 method based on Euclidean distance. As shown in Figure 5C, there was a remarkable difference in color between the control and the CA exposure groups. More detailed information about DMs is provided in Table S1.

**Figure 6:**
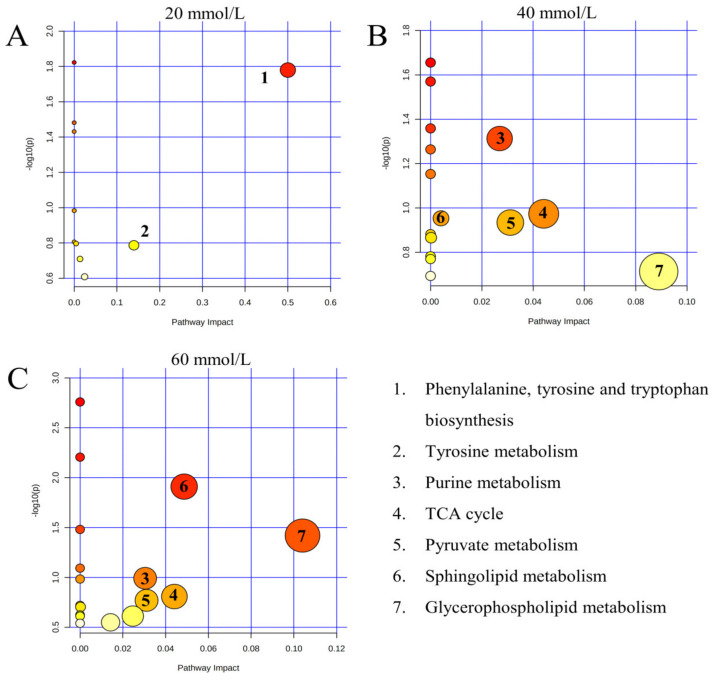
Metabolic pathway analysis of crucian carp kidney under different concentrations of carbonate alkali exposure. (**A**) The metabolic pathway changes of crucian carp kidney after 20 mmol/L NaHCO_3_ exposure. (**B**) The metabolic pathway changes of crucian carp kidney after 40 mmol/L NaHCO_3_ exposure. (**C**) The metabolic pathway changes of crucian carp kidney after 60 mmol/L NaHCO_3_ exposure.

**Figure 7:**
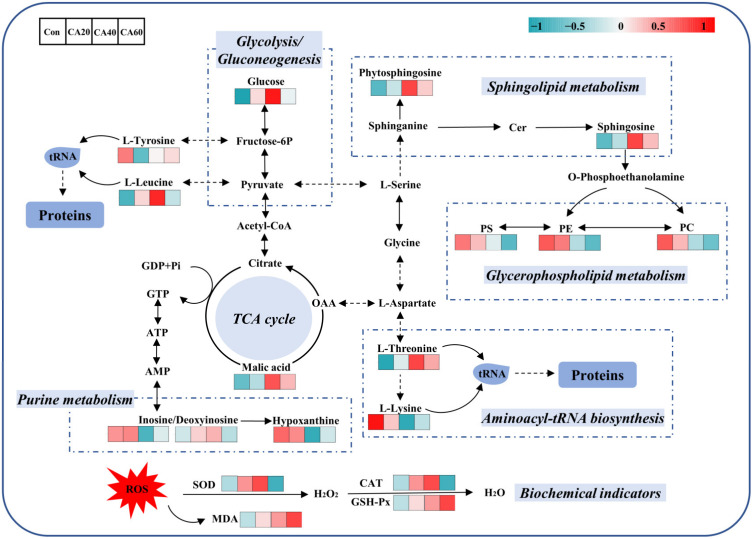
The physiological and metabolic changes of crucian carp kidney under carbonate alkalinity exposure. Con represents the freshwater control group, CA20 represents the 20 mmol/L NaHCO_3_ exposure group, CA40 represents the 40 mmol/L NaHCO_3_ exposure group, and CA60 represents the 60 mmol/L NaHCO_3_ exposure group.

The metabolome view in Figure 6 showed all matched pathways in each exposure group with the most impacted pathways highlighted in red, which were generated by MetaboAnalyst 5.0. Detailed information about all matched metabolic pathways under various concentrations of carbonate alkalinity exposure is provided in the Supplementary Material Table S2. Based on impact scores from the pathway topology analysis, *p*-value from the pathway enrichment analysis (student *t*-test), and the pathway physiological function, the results showed that 20 mmol/L NaHCO_3_ exposure concentration had little effect on the kidney metabolism but mainly changed tyrosine metabolism (*p* > 0.05) and phenylalanine, tyrosine, and tryptophan biosynthesis (*p* < 0.05). There was a significant increase in the number of affected metabolic pathways with the further increase in NaHCO_3_ exposure concentration, including purine metabolism, TCA cycle, pyruvate metabolism, sphingolipid metabolism and glycerophospholipid metabolism. Ultimately, a network diagram was drawn based on the results of the biochemical and metabonomic studies, which provided visual information concerning the physiological and metabolic changes occurring in the crucian carp kidney under various exposure concentrations of carbonate alkalinity (Figure 7).

## 4. Discussion

### 4.1. Carbonate Alkaline Stress-Induced Oxidative Damage in Kidney

Aquatic animals have developed a battery of defense mechanisms against oxidative stress induced by environmental stress [27]. The antioxidant system is responsible for maintaining reactive oxygen species (ROS) homeostasis when the organism is under normal physiological conditions [28]. However, the excessive accumulation of ROS occurs when ROS production exceeds the capacity of the antioxidant system or when the ROS detoxifying system is compromised, which causes severe damage to the structure and function of the cell membrane leading eventually to cell death [21,29]. Numerous previous studies have demonstrated that saline-alkaline exposure leads to the excessive accumulation of ROS and causes oxidative stress in aquatic animals [30]. Antioxidant enzymes are important components of the fish’s antioxidant system, and the SOD–CAT–GSH-Px cascade serves as the first line of defense against ROS [31]. SOD catalyzes the decomposition of harmful superoxide anion radicals into hydrogen peroxide, which is then further completely decomposes into harmless water by CAT and GSH-Px, thus protecting the cell from oxidative stress damage. In the present study, compared to the control group, the activities of SOD and CAT in the kidney were greatly increased at 20 and 40 mmol/L NaHCO_3_ and sharply decreased at 60 mmol/L NaHCO_3_. Notably, with the increase in carbonate alkalinity exposure concentration, SOD and CAT activities in the kidney initially increased and then decreased, while GSH-Px activity kept increasing. The above results showed that fish can effectively cope with CA stress by regulating antioxidant enzyme activity [32]. However, when SOD and CAT were further weakened or even ruined in long-term high-concentration carbonate alkalinity, enhanced kidney GSH-Px activity would help clear accumulated H_2_O_2_ and hydroxylated compounds to protect the renal tissues from oxidative stress damage [4,21]. The MDA content is a crucial parameter to reflect the degree of lipid peroxidation and tissue damage caused by ROS [33]. MDA content in kidney were increased significantly with the increase in exposure concentration, suggesting CA stress induced lipid peroxidation in the kidney of crucian carp. This finding is consistent with previous studies on lipid peroxidation in fish kidney cells caused by carbonate alkali exposure [34,35].

Oxidative stress induced by environmental stress can cause changes in the tissue morphology of aquatic animals, which inevitably leads to changes in function [5,36,37,38]. The changes in the renal tissue of crucian carp induced by CA exposure with different concentrations were proven by histopathology in the present study. The renal histopathology showed that with the increase in carbonate alkali exposure concentration, there was renal tubular epithelial cell degranulation, swelling and autolysis, renal tubular obstruction, glomerular atrophy and swelling, and interstitial cell proliferation, swelling and necrosis, which was consistent with previous research [19,34,35]. BUN and CREA are used as critical markers of impaired renal function [39]. Our study found that the levels of BUN and CREA in the serum of crucian carp greatly increased in a concentration-dependent manner with CA exposure, which might result from the damage of renal tubules and glomeruli caused by carbonate alkali stress.

### 4.2. Effect of Carbonate Alkaline Stress on Kidney Metabolism

#### 4.2.1. Amino Acid Metabolism

Amino acids are involved in various biological processes such as nitrogen metabolism, protein synthesis, energy metabolism and immune regulation [40]. It has been proven that the conversion of ammonia into amino acids to reduce ammonia accumulation is one of the important survival strategies for fish to adapt to the saline-alkali environment [41,42,43]. In this study, the levels of L-threonine and L-leucine increased significantly under stress conditions compared to the control, while L-tyrosine and L-lysine decreased. L-threonine, as an important restrictive amino acid in fish immunoglobulin, promotes the production of immunoglobulin lymphocytes and antibodies [44]. L-leucine and L-lysine have physiological functions of regulating fish growth, metabolism and immune response [45,46]. L-tyrosine is an essential substrate of melanin synthesis, and melanin plays an important role in wound healing and antimicrobial host defense responses to promote an immune response in aquatic animals [47,48]. Our findings clearly revealed the disorder of amino acid metabolism in the crucian carp kidney could be induced at a low concentration of NaHCO_3_ (20 mmol/L), including phenylalanine, tyrosine and tryptophan biosynthesis, tyrosine metabolism and valine, leucine and isoleucine biosynthesis (Table S2). At the same time, the kidney could regulate amino acid anabolism to produce immune-related metabolites for immune regulation to alleviate the damage caused by CA stress. In addition, metabolic pathway analysis showed that these affected amino acids were also involved in aminoacyl-tRNA biosynthesis. Amino acids combined with tRNA are transported into ribosomes for protein synthesis [49]. The accumulation of amino acids in the crucian carp kidney increased with an increase in carbonates, implying that increasing inhibition of protein synthesis in the crucian carp kidney was seen with increasing concentration of carbonate alkali. Environmental stress causes amino acid metabolism disorder in fish, which in turn interferes with aminoacyl-tRNA biosynthesis and protein digestion and absorption, which has been found in olive flounder (*Paralichthys olivaceus*) [40], swordtail fish (*Xiphophorus helleri*) [50], and common carp (*Cyprinus carpio*) [51]. Collectively, based on the analysis above, crucian carp could suppress protein and amino acid catabolism of the kidney to reduce endogenous ammonia production for the adaptations of the saline-alkaline environment.

#### 4.2.2. Lipid Metabolism

Lipid metabolism is one of the most critical regulatory pathways in the adaptation of aquatic animals to environmental stress [52]. Numerous studies have proved that exposure to saline-alkali can disrupt the lipid metabolism of aquatic animals [53,54]. Liu et al. [3] reported that carbonate alkali exposure induced significant changes in metabolites and gene expression levels in lipid metabolism, including glycerophospholipid metabolism, sphingolipid metabolism and arachidonic acid metabolism in crucian carp. Song et al. [55] showed that Nile tilapia (*Oreochromis niloticus*) were more inclined to use lipid metabolism for energy supply in adapting to high concentrations of saline-alkaline water. Zhao et al. [56] showed that many differentially expressed genes changed significantly in the arachidonic acid metabolism of *Leuciscus waleckii* under a highly alkaline environment. In our study, lipid metabolism in the kidney of crucian carp was not affected by 20 mmol/L NaHCO_3_ stress. With increasing concentrations of NaHCO_3_, multiple pathways related to lipid metabolism in the kidney of crucian carp began to change, including glycerophospholipid metabolism, sphingolipid metabolism, arachidonic acid metabolism, linoleic acid metabolism, and α -linolenic acid metabolism. It was noted that glycerophospholipid metabolism and sphingolipid metabolism were significantly affected at 60 mmol/L NaHCO_3_ (*p* < 0.05).

Glycerophospholipids and sphingomyelins are not only indispensable components of biological cell membranes but also key participants in substance transportation, energy conversion and the signal transduction of organisms [57]. Phosphatidylcholine (PC), phosphatidylethanolamine (PE), and phosphatidylserine (PS) are glycerophospholipid pathway metabolites that not only keep the stability of cell membranes against stress to relieve cell injury but also maintain cellular energy homeostasis [58]. In the process of sphingolipid metabolism, sphingosine (Sph) and phytosphingosine (Phs) have high biological activity, which can directly participate in inflammatory reactions and signal transduction pathways such as cell proliferation, differentiation, and the apoptosis of various cell types [59]. In this study, compared with the control group, the levels of PC, PE, and PS were significantly decreased with increasing salinity-alkalinity stress concentration (*p* < 0.05), while the levels of Sph and Psh were increased to different degrees in the exposure group, glycerophospholipid metabolism and sphingolipid metabolism involved in these metabolites emerged as the most significantly disturbed pathway at 60 mmol/L NaHCO_3_ (*p* < 0.05). The findings indicated that long-term carbonate alkalinity stress of 60 mmol/L could further exacerbate the damage to the renal cell membrane structure, cause disorders of glycerophospholipid metabolism and sphingolipid metabolism, and induce inflammatory reaction and apoptosis, which in turn cause damage to the fish at both the cellular level and tissue architecture.

#### 4.2.3. Purine Metabolism

Purine nucleotides naturally exist in the cellular environment, they are not only structural components of DNA and RNA molecules but also cofactors mediating cell signal transduction in many metabolic processes and the source of the energy provider adenosine triphosphate (ATP) [60]. Purine metabolism is one of the metabolic pathways related to the immune system in organisms, which serves a protective role in ameliorating oxidative stress and tissue injury in multiple organs [61]. In this study, differential metabolites related to purine metabolism were changed with different concentrations of NaHCO_3_, including inosine, deoxyinosine, and hypoxanthine. Specifically, the levels of inosine, hypoxanthine and deoxyinosine were not significantly changed at the exposure concentrations of 20 mmol/L NaHCO_3_; they decreased significantly at the exposure concentrations of 40 and 60 mmol/L NaHCO_3_. In the process of purine metabolism, adenosine and deoxyadenosine are hydrolyzed by adenosine deaminase into corresponding inosine and deoxyinosine and further transformed into hypoxanthine, which can be irreversibly converted into uric acid, producing excessive reactive oxygen species (ROS) and hydrogen peroxide [62,63]. Inosine has potent immunomodulatory and neuroprotective effects [64]. Deoxyinosine is considered to be a potential cancer biomarker [65]. Hypoxanthine is a product of apoptotic and lysed DNA metabolism, with metabolite levels that reflect cell cycle or cell death activity [66]. The above analysis results showed that when the concentration of carbonate alkalinity reached 40 mmol/L, the purine metabolism in the crucian carp kidney would be disordered, which could lead to an imbalance in the immune system. This finding concerning disorders of purine metabolism in fish induced by carbonate exposure was similar to previous research on fish purine metabolism disorders induced by environmental exposure such as hypoxia stress [67], ammonia stress [68] and cold stress [69].

#### 4.2.4. Energy Metabolism

What is critical for the survival of aquatic organisms under environmental stress is the mobilization of energy reserves [70]. Glucose is one of the sources of energy supply in organisms. During stress, glycolysis and gluconeogenesis occur in the fish body to provide glucose and energy [71]. In this study, the glycolytic pathway in the crucian carp kidney was slightly affected at the exposure concentrations of 20 mmol/L NaHCO_3_ (*p* > 0.05). When the exposure concentration increased to 40, 60 mmol/L, not only the glycolytic pathway but also the TCA cycle and pyruvate pathway were affected. In addition, the levels of D-glucose were greater in the CA exposure groups than in the control group, and initially increased and then decreased with 40 mmol/L NaHCO_3_ being the turning point. Moreover, the content of L-malic acid, the intermediate of the TCA cycle, was found to accumulate in the kidney at the exposure concentration of 40, 60 mmol/L NaHCO_3_. The results of the above studies indicated that crucian carp could produce a large amount of glucose to maintain energy homeostasis by regulating the glycolysis/gluconeogenesis pathway at 20 mmol/L NaHCO_3_. As the concentration of NaHCO_3_ increased above 40 mmol/L, the glycolysis/gluconeogenesis pathway could no longer meet the high energy demand of crucian carp for saline-alkali adaptation. At this time, the TCA cycle of the crucian carp kidney was regulated to provide more energy. The TCA cycle is the central hub that connects carbohydrate, lipid, and amino acid metabolism [72]. Previous research has shown that inflammation can disrupt the TCA cycle, and TCA cycle intermediates play a crucial role in regulating the cellular immune response [73]. As an intermediate product of the TCA cycle, L-malic acid has the function of enhancing the immune response of fish [74]. The increased malic acid in the kidney indicated that the TCA cycle plays a key role in the inflammatory response induced by saline-alkali stress.

## 5. Conclusions

In summary, we used integrated analysis of the metabolomics, histopathology, and biochemical indicators to investigate the effects of saline-alkaline stress on the renal structure and function, antioxidant capacity and metabolism of the crucian carp kidney. Saline-alkaline stress could trigger oxidative stress for the imbalance between excessive ROS production and antioxidant systems, which causes severe damage to the renal structure and function. Simultaneously, crucian carp could suppress protein and amino acid catabolism of the kidney to reduce endogenous ammonia for survival in the saline-alkaline environment. After long-term exposure to saline-alkaline conditions, multiple metabolic pathways in the kidney of crucian carp were seriously disturbed including aminoacyl-tRNA biosynthesis, purine metabolism, glycerophospholipid metabolism, sphingolipid metabolism, and glycolysis/gluconeogenesis in the crucian carp kidney, which induced immune inflammatory reactions and apoptosis. The findings in this study will facilitate the understanding of the metabolic regulation of crucian carp in response to carbonate alkali stress at the cellular level and provide a theoretical basis for cultivating excellent salt-tolerant fish species and carrying out freshwater fish breeding activities in saline-alkali water.

## Data Availability

Data available on request due to privacy.

## Supplementary Materials

The following supporting information can be downloaded at: https://www.mdpi.com/article/10.3390/metabo13020159/s1, Table S1: The different metabolites in the kidney of crucian carp under different exposure concentrations of carbonate alkali stress; Table S2: Changes of kidney metabolic pathway of crucian carp under different exposure concentrations of carbonate alkali stress.
