# Supplementary material for: Effects of Saline-Alkaline Stress on Metabolome, Biochemical Parameters, and Histopathology in the Kidney of Crucian Carp (Carassius auratus)
Source: Metabolites. 2023 Jan 20;13(2):159. doi: 10.3390/metabo13020159 (PMC9966543; doi:10.3390/metabo13020159)
Supplement: Supplementary file 1 [file metabolites-13-00159-s001.zip › metabolites-2120768-SI.pdf]

metabolites

Supplementary Materials

Effects of Saline-Alkaline Stress on Metabolome, Biochemical Parameters, and Histopathology in the Kidney of Crucian Carp (*Carassius Auratus*)

Lu Ding [1,2,†], Yingjie Liu [1,2,†], Xiaofeng Wei [1,3], Chuanye Geng [1,2], Wenzhi Liu [1,2], Lin Han [1,2], Fangying Yuan [1,4], Peng Wang [1] and Yanchun Sun[1,2,3,4,*]

1 Laboratory of Quality & Safety Risk Assessment for Aquatic Products, Heilongjiang River Fisheries Research Institute, Chinese Academy of Fishery Sciences, Ministry of Agriculture and Rural Areas, Harbin 150070, China
2 Department of Food Science and Engineering, College of Food Science and Technology, Shanghai Ocean University, Shanghai 201306, China
3 Department of Food Science and Engineering, College of Food Science and Engineering, Dalian Ocean University, Dalian 116023, China
4 Department of Chemical Engineering and Technology, College of Materials and Chemical Engineering, Harbin University of Science and Technology, Harbin 150080, China
* Correspondence: sunyanchun@hrfri.ac.cn
† These authors contributed equally to this work.

Reprint requests to Tel: +86-0451-84861316. Fax: +86 0451 84604803.

Text captions:

Text S1. Metabolomics Analysis.

Table captions:

Table S1. The different metabolites in the kidney of crucian carp under different exposure concentrations of carbonate alkali stress.

Table S2. Changes of kidney metabolic pathway of crucian carp under different exposure concentrations of carbonate alkali stress.

Metabolomics Analysis

The kidney tissues (90 mg) were homogenized with 1200 μL pre-cooled extraction sol-vent (methanol: water = 4:1, v/v) at −20 °C, 60 Hz for 2 min, ultrasonicated at 4 °C for 30 min, set at −20 °C for 30 min, and centrifuged at 4 °C, 13,000 rpm for 15 min. The supernatant (600 μL) was filtered into a liquid chromatography glass vial through 0.22 μm organic phase filter membrane for UPLC-QTOF/MS analysis. In addition, equal amounts of samples were mixed to prepare quality control (QC) samples to monitor errors caused by experimental operation and instruments.

The chromatographic separation was performed on the pre-column of Waters Acquity UPLC BEH C18 (2.1 mm × 100 mm, 1.7 μm) and AQUITY UPLC BEH C18 (2.1 mm × 5 mm, 1.7μm). The mobile phase A was 0.1% formic acid in water (v/v), and the mobile phase B was 0.1% formic acid in acetonitrile (v/v). The gradient elution procedure was performed as follows: 0~2 min, 5%~20% B; 2~3min, 20%~60% B; 3~11min, 60%~80% B; 11~12 min, 80%~100% B; 12~13 min, 100%~100% B; 13~13.1 min, 100%~5% B; 13.1~15 min, 5%~5% B. The column and autosampler tray temperature were maintained at 30 °C and 4 °C, respectively. The flow rate was set to 0.3 mL/min, and the injection volume was set to 10 μL.

Mass spectrometry analysis used a Triple TOF 5600+ system with an electrospray ionization (ESI) source (SCIEX, Framingham, MA, USA). The TOF/MS conditions were as follows: ion spray voltage was set to 5000 V (ESI+) or 4500 V (ESI−), ion source temperature

was 550 °C, declustering potential was ±80 V, collision energy was ±35 eV, collision energy spread was ±15 eV. Nitrogen was used as an atomizing gas and auxiliary gas. The pressure of Gas1 and Gas2 was 55 psi, and the curtain gas was 35 psi. The TOF-MS scan was configured to acquire the MS fragment ions, and the mass scan range was 100–1200 Da. The information-dependent acquisition (IDA) was configured to ac-quire the MS/MS fragment ions and the mass scan range was 50–1200 Da.

Table S1. The different metabolites in the kidney of crucian carp between carbonate alkalinity stress groups and freshwater control group.

RT (min)	Compound ID	Description	Formula	M/Z	Adducts	Mass Error (ppm)	VIP-Value	*p*-Value
**CA20 vs Con**								
0.87	HMDB0000095	Cytidine monophosphate	C9H14N3O8P	322.0453	M-H	2.12	1.08	0.00181912
1.30	HMDB0000157	Hypoxanthine	C5H4N4O	137.0451	M+H	−4.72	1.42	0.00318102
2.39	HMDB0000158	L-Tyrosine	C9H11NO3	146.0594	M+H-2H2O	−3.57	1.66	0.000796661
3.41	HMDB0000687	L-Leucine	C6H13NO2	114.0907	M+H-H2O	−4.99	5.19	0.00248894
3.94	HMDB0011603	4-(Methylnitrosamino)-1-(3-pyridyl)-1-butanone	C10H13N3O2	415.2078	2M+H	−2.50	1.08	0.0020299
7.40	HMDB0000122	D-Glucose	C6H12O6	383.1153	2M+Na	−1.80	1.12	3.33418×10^{-5}
**CA40 vs Con**								
1.30	HMDB0000157	Hypoxanthine	C5H4N4O	137.0451	M+H	−4.72	2.78	0.000118691
1.30	HMDB0000195	Inosine	C10H12N4O5	267.0746	M-H	4.21	1.49	0.00567422
1.52	HMDB0000182	L-Lysine	C6H14N2O2	129.1016	M+H-H2O	−4.44	1.11	1.18792×10^{-6}
14.09	HMDB0007889	PC(14:0/22:4(7Z,10Z,13Z,16Z))	C44H80NO8P	782.5703	M+H	1.08	2.71	0.00159672
14.09	HMDB0007988	PC(16:0/22:4(7Z,10Z,13Z,16Z))	C46H84NO8P	832.5815	M+H,	−1.70	1.27	0.000535817
14.10	HMDB0003447	Tryptophanol	C10H11NO	184.0731	M+Na	−1.17	2.14	0.00166271
14.10	HMDB0008908	PE(15:0/22:1(13Z))	C42H82NO8P	760.5854	M+H	0.40	1.33	0.00319517
14.12	HMDB0008842	PE(14:0/22:1(13Z))	C41H80NO8P	790.5418	M-H,	4.69	1.36	0.0249602
3.41	HMDB0000687	L-Leucine	C6H13NO2	114.0907	M+H-H2O	−4.99	5.34	1.28311×10^{-5}
4.25	HMDB0004610	Phytosphingosine	C18H39NO3	318.2997	M+H	−1.78	1.33	0.000111784
7.40	HMDB0000122	D-Glucose	C6H12O6	383.1153	2M+Na	−1.80	1.08	3.2931×10^{-9}
7.60	HMDB0000156	L-Malic acid	C4H6O5	135.0284	M+H	−2.71	1.01	0.000466994
**CA60 vs Con**								
0.84	HMDB0000071	Deoxyinosine	C10H12N4O4	527.1591	2M+Na	−3.63	1.86	0.000202808
0.85	HMDB0000167	L-Threonine	C4H9NO3	84.0439	M+H-2H2O	−4.14	1.17	0.000319363
0.97	HMDB0000290	UDP-N-acetyl-alpha-D-glucosamine	C17H27N3O17P2	606.0766	M-H	3.73	1.58	0.0328581
1.30	HMDB0000157	Hypoxanthine	C5H4N4O	137.0451	M+H	−4.72	3.10	0.00143541
1.52	HMDB0000182	L-Lysine	C6H14N2O2	129.1016	M+H-H2O	−4.44	1.38	0.000108234
12.53	HMDB0007991	PC(16:0/22:6(4Z,7Z,10Z,13Z,16Z,19Z))	C46H80NO8P	828.5493	M+H	−4.30	1.28	0.0027635
14.09	HMDB0007889	PC(14:0/22:4(7Z,10Z,13Z,16Z))	C44H80NO8P	782.5703	M+H	1.08	4.58	0.00134681
14.09	HMDB0112383	PS(18:0/22:5(4Z,7Z,10Z,13Z,16Z))	C46H80NO10P	802.5368	M+Na	−1.55	1.61	0.005765
14.09	HMDB0007988	PC(16:0/22:4(7Z,10Z,13Z,16Z))	C46H84NO8P	832.5815	M+H	−1.70	2.51	1.29691×10^{-5}
14.09	HMDB0008452	PC(20:4(5Z,8Z,11Z,14Z)/22:6(4Z,7Z,10Z,13Z,16Z,19Z))	C50H80NO8P	854.5684	M+H	−1.20	2.20	4.41387×10^{-6}
14.10	HMDB0003447	Tryptophanol	C10H11NO	184.0731	M+Na	−1.17	4.17	0.000108405
14.10	HMDB0010382	LysoPC(16:0)	C24H50NO7P	478.3292	M+H-H2O	−0.10	1.65	6.18578×10^{-5}
14.10	HMDB0008908	PE(15:0/22:1(13Z))	C42H82NO8P	760.5854	M+H	0.40	2.04	0.00599753
14.12	HMDB0007940	PC(15:0/18:2(9Z,12Z))	C41H78NO8P	764.5262	M-H	4.24	1.42	5.66491×10^{-6}
14.12	HMDB0008842	PE(14:0/22:1(13Z))	C41H80NO8P	790.5418	M-H	4.69	3.18	0.000129376
3.41	HMDB0000687	L-Leucine	C6H13NO2	114.0907	M+H-H2O	−4.99	3.86	0.0388549
4.25	HMDB0004610	Phytosphingosine	C18H39NO3	318.2997	M+H	−1.78	1.66	0.000584802
4.28	HMDB0000252	Sphingosine	C18H37NO2	300.2888	M+H	−3.06	1.28	0.000175917
7.40	HMDB0000122	D-Glucose	C6H12O6	383.1153	2M+Na	−1.80	1.12	0.00025648
7.60	HMDB0000156	L-Malic acid	C4H6O5	135.0284	M+H	−2.71	1.34	0.00119402

Note: Con represents the freshwater control group, CA20 represents the 20 mmol/L $NaHCO_3$ exposure group, CA40 represents the 40 mmol/L $NaHCO_3$ exposure group, and CA60 represents the 60 mmol/L $NaHCO_3$ exposure group.

Table S2. Changes of kidney metabolic pathway of crucian carp under different exposure concentrations of carbonate alkali stress.

Pathway Name	*p*-Value	−log(p)	Impact
**CA20 vs Con**			
Aminoacyl-tRNA biosynthesis	0.015046	1.8226	0
Phenylalanine, tyrosine and tryptophan biosynthesis	0.016614	1.7795	0.5
Valine, leucine and isoleucine biosynthesis	0.032998	1.4815	0
Phenylalanine metabolism	0.032998	1.4815	0
Ubiquinone and other terpenoid-quinone biosynthesis	0.037058	1.4311	0
Glycolysis/Gluconeogenesis	0.10394	0.98322	2.10×10^{-4}
Valine, leucine and isoleucine degradation	0.15607	0.80669	0
Pyrimidine metabolism	0.15969	0.79672	0.00442
Tyrosine metabolism	0.1633	0.787	0.13972
Metabolism of xenobiotics by cytochrome P450	0.19523	0.70945	0.0137
Purine metabolism	0.24618	0.60874	0.02445
**CA40 vs Con**			
Linoleic acid metabolism	0.022106	1.6555	0
Aminoacyl-tRNA biosynthesis	0.02691	1.5701	0
Valine, leucine and isoleucine biosynthesis	0.043784	1.3587	0
Purine metabolism	0.048643	1.313	0.02694
Biotin metabolism	0.054464	1.2639	0
alpha-Linolenic acid metabolism	0.070288	1.1531	0
Citrate cycle (TCA cycle)	0.10631	0.97341	0.04412
Sphingolipid metabolism	0.11136	0.95327	0.00406
Pyruvate metabolism	0.11638	0.93412	0.0311
Lysine degradation	0.13129	0.88176	0
Glycolysis/Gluconeogenesis	0.13622	0.86577	2.10×10^{-4}
Glyoxylate and dicarboxylate metabolism	0.16524	0.78189	0
Arachidonic acid metabolism	0.16999	0.76958	0
Glycerophospholipid metabolism	0.1934	0.71354	0.08898
Valine, leucine and isoleucine degradation	0.2026	0.69335	0
**CA60 vs Con**			
Valine, leucine and isoleucine biosynthesis	0.0017418	2.759	0
Aminoacyl-tRNA biosynthesis	0.0062283	2.2056	0
Sphingolipid metabolism	0.012297	1.9102	0.04868
Linoleic acid metabolism	0.033021	1.4812	0
Glycerophospholipid metabolism	0.038038	1.4198	0.10403
Biotin metabolism	0.080682	1.0932	0
Purine metabolism	0.10194	0.99166	0.03044
alpha-Linolenic acid metabolism	0.1037	0.98424	0
Citrate cycle (TCA cycle)	0.15536	0.80867	0.04412
Pyruvate metabolism	0.16961	0.77056	0.0311
Lysine degradation	0.19057	0.71995	0
Glycolysis/Gluconeogenesis	0.19745	0.70455	2.10×10^{-4}
Glyoxylate and dicarboxylate metabolism	0.23761	0.62414	0
Arachidonic acid metabolism	0.24412	0.6124	0
Glycine, serine and threonine metabolism	0.24412	0.6124	0.02466
Amino sugar and nucleotide sugar metabolism	0.28213	0.54955	0.01426
Valine, leucine and isoleucine degradation	0.28829	0.54017	0

Note: Con represents the freshwater control group, CA20 represents the 20 mmol/L $NaHCO_3$ exposure group, CA40 represents the 40 mmol/L $NaHCO_3$ exposure group, and CA60 represents the 60 mmol/L $NaHCO_3$ exposure group.
